# Effectiveness and Safety of Topically Applied Tranexamic Acid with Epinephrine in Surgical Procedures: A Systematic Review

**DOI:** 10.3390/ebj6030052

**Published:** 2025-09-22

**Authors:** Hedieh Keshavarz, Weber Wei Chiang Lin, Shawn Dodd, Janice Y. Kung, Joshua N. Wong

**Affiliations:** 1 Faculty of Medicine and Dentistry; University of Alberta, Edmonton, AB T6G 1C9, Canada; 2 Division of Plastic Surgery, Department of Surgery, University of Alberta, Edmonton, AB T6G 1C9, Canada; 3 Geoffrey & Robyn Sperber Health Sciences Library, University of Alberta, Edmonton, AB T6G 1C9, Canada; janice.kung@ualberta.ca

**Keywords:** tranexamic acid, perioperative bleeding, surgical hemostasis

## Abstract

Background: Topical tranexamic acid (TXA), often combined with epinephrine, is used to reduce perioperative bleeding. This systematic review evaluates the safety and effectiveness of this combination across surgical procedures. Methods: A comprehensive search of eight databases was conducted from inception to 26 June 2025. Studies were eligible if they compared topically or locally applied TXA with epinephrine to epinephrine alone in surgical patients. Animal studies, case reports, non-English publications, and studies without comparators were excluded. Screening, data extraction, and risk of bias assessments followed PRISMA guidelines. Results: Ten studies met inclusion criteria (four randomized and six non-randomized), covering burn surgery, rhytidectomy, liposuction, septoplasty, endoscopic sinus surgery, dacryocystorhinostomy, and joint arthroplasty. TXA was applied topically or via tumescent infiltration. Most studies reported reduced intraoperative blood loss, improved surgical field visibility, lower drain output, shorter hemostasis time, and reduced transfusion rates. No increase in thromboembolic or major complications was observed. Conclusion: The combination of TXA and epinephrine appears safe and maybe effective for perioperative bleeding control. However, heterogeneity in dosing and outcomes limits generalizability. Further research is needed to standardize protocols and confirm long-term safety.

## 1. Introduction

Perioperative bleeding remains a significant clinical concern and is associated with increased morbidity, mortality, and healthcare resource utilization [1]. Hemostatic agents are widely used across surgical specialties and are considered a standard component of perioperative care, particularly in major procedures [2]. The prevention of intraoperative and postoperative bleeding begins at the time of incision and continues throughout the procedure. Consistent, immediate, and meticulous hemostasis is essential, as even minor bleeding can accumulate to significant losses during longer surgeries [3,4].

A variety of strategies are employed to minimize perioperative bleeding. With a growing number of patients presenting with increased bleeding risk—often due to the widespread use of antiplatelet and anticoagulant therapies—as well as surgeries with a high risk of bleeding, comprehensive preoperative risk stratification has become increasingly important [5,6,7]. Current best practices emphasize the use of standardized tools, such as structured questionnaires assessing bleeding and drug history, which are considered more effective than routine coagulation screening tests in identifying patients at risk [8]. Once bleeding risk is evaluated, targeted perioperative interventions may include the use of various hemostatic agents [9]. Topical therapies such as vasoconstrictors, antifibrinolytics, and procoagulant factors have long played a central role due to their localized action and favorable safety profile [7,10,11].

Tranexamic acid (TXA) is a synthetic lysine analog that exerts its antifibrinolytic effect by competitively inhibiting the activation of plasminogen to plasmin. By blocking lysine-binding sites on plasminogen, TXA prevents fibrin degradation and promotes stabilization of the fibrin clot formed during secondary hemostasis [12,13]. Intravenous TXA has been shown to significantly reduce blood loss in various surgical settings but concerns about systemic absorption and thromboembolic events have limited its use in certain populations. Consequently, topical or locally infiltrated TXA has gained increasing attention as a strategy to achieve localized hemostasis with reduced systemic exposure [14,15,16,17].

Epinephrine, an adrenergic vasoconstrictor, is commonly used via local infiltration or topical application to constrict peripheral blood vessels, thereby reducing intraoperative bleeding and prolonging the duration of local anesthetic effect [18,19]. When used in combination, TXA and epinephrine target two distinct mechanisms of bleeding control—coagulation stabilization and vasoconstriction—offering a synergistic approach to perioperative blood loss management. This dual-action strategy may be particularly advantageous in surgeries where minimizing systemic drug exposure is critical or where bleeding control is technically challenging [18,19,20].

Despite growing interest in the use of topical tranexamic acid, particularly in combination with epinephrine, the literature remains heterogeneous in terms of surgical indications, dosing strategies, delivery methods, and outcome measures. To date, no systematic review has comprehensively evaluated the use of topically or locally applied tranexamic acid in combination with epinephrine across different surgical disciplines. This review aims to synthesize the available evidence on the safety and effectiveness of this combined approach, with a focus on outcomes such as intraoperative and postoperative blood loss, complications, and overall surgical utility.

## 2. Methods

### 2.1. Study Design

A systematic review was conducted in accordance with the Preferred Reporting Items for Systematic Reviews and Meta-Analyses (PRISMA) guidelines [21]. The study protocol was registered in OSF retrospectively on 29 August 2025. (DOI: https://doi.org/10.17605/OSF.IO/WNFQK).

### 2.2. Information Sources and Search Strategy

The literature search was conducted by a medical librarian (JYK) in collaboration with the research team, following the Preferred Reporting Items for Systematic Reviews and Meta-Analyses (PRISMA) guidelines. Comprehensive searches were developed and executed across multiple databases, including Ovid MEDLINE, Ovid Embase, CINAHL, the Cochrane Library (via Wiley), Scopus, Web of Science Core Collection, and ProQuest Dissertations & Theses Citation Index. Searches were first conducted on 10 March 2023, and subsequently updated on 26 June 2025. To identify studies evaluating the use of topically applied tranexamic acid (TXA) and epinephrine for bleeding management in burn surgeries, relevant keywords and controlled vocabulary were carefully selected. No language or date restrictions were applied. The full search strategy is provided in Appendix A. In addition to database searches, the research team searched trial registries (e.g., ClinicalTrials.gov) and screened the first 200 results from Google Scholar, consistent with prior evidence showing substantial overlap with indexed databases [22]. Bibliographies of included studies were also reviewed to identify additional relevant publications.

### 2.3. Eligibility Criteria

We included studies evaluating the use of tranexamic acid (TXA) applied topically or locally in combination with epinephrine in surgical patients of any age. Eligible studies reported at least one perioperative outcome, including intraoperative or postoperative blood loss, drain output, ecchymosis, transfusion rates, operative time, or postoperative complications. We considered randomized controlled trials, prospective or retrospective cohort studies, and within-subject comparative designs. Studies without a comparator arm were included if they reported outcomes clearly following TXA plus epinephrine administration. We excluded studies not published in English, those evaluating TXA without concurrent epinephrine, and studies using only systemic TXA administration. Studies focused on oral and maxillofacial procedures were excluded due to differences in surgical context, outcome reporting, and intervention delivery, which limited comparability with other surgical specialties. Case reports, editorials, single-arm studies without outcome data, and narrative reviews were also excluded. No restrictions were applied regarding date or geographic location.

### 2.4. Study Selection and Data Collection

All records retrieved from the database search were uploaded to Covidence for de-duplication, screening, and full-text review. Two independent reviewers conducted title and abstract screening, followed by full-text assessment of potentially eligible articles. Disagreements were resolved through discussion and consensus. A PRISMA flow diagram was generated to summarize the screening and selection process, including reasons for exclusion at the full-text stage. Data from eligible studies were extracted using a structured Excel spreadsheet developed for this review. Extracted variables included study design, surgical procedure, sample size, TXA dose and delivery method, presence of a comparator group, use of epinephrine, reported outcomes, and complications. A second reviewer independently verified the extracted data for accuracy and completeness. Discrepancies were addressed through discussion, and Supplementary Materials were reviewed when available to ensure data completeness.

### 2.5. Risk of Bias Assessment

Risk of bias was assessed using standardized tools appropriate to study design. For randomized controlled trials, the Cochrane Risk of Bias 2 (RoB-2) tool was used [23]. For non-randomized studies, the Risk of Bias in Non-randomized Studies of Interventions (ROBINS-I) tool was applied [24]. Each study was evaluated across multiple domains including bias due to confounding, selection of participants, classification of interventions, deviations from intended interventions, missing data, measurement of outcomes, and selection of reported results. Judgments were categorized as low, moderate, serious, or critical risk of bias. Risk of bias assessments were recorded in dedicated excel worksheets.

### 2.6. Data Synthesis

Given the heterogeneity in surgical procedures, study designs, dosing regimens, and outcome measures, a meta-analysis was not feasible. Instead, a narrative synthesis was performed to summarize study characteristics and findings. Studies were grouped thematically based on the type of surgical procedure and were compared in terms of TXA administration protocols, presence of a comparator group, and reported outcomes such as intraoperative blood loss, ecchymosis, drain output, and complications. Findings were summarized qualitatively to identify patterns, consistencies, and limitations across the included studies.

## 3. Results

**Figure 1:**
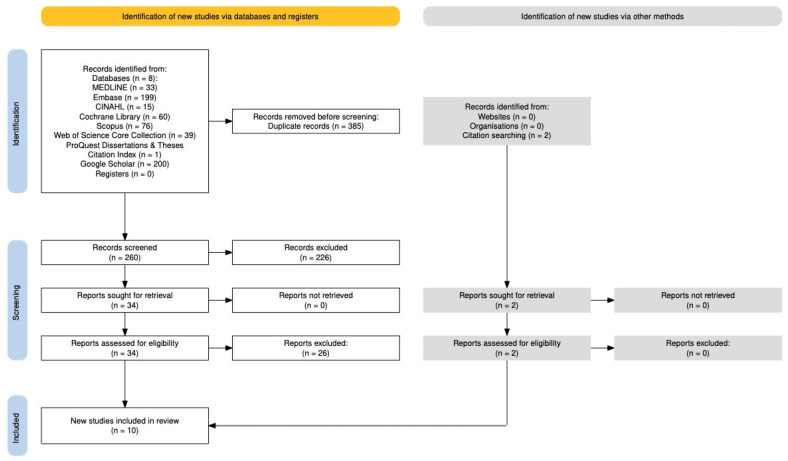
PRISMA 2020 flow diagram outlining the study selection process. 8 studies met inclusion criteria through direct screening, and 2 additional studies were identified from relevant systematic reviews, resulting in eight studies included in the final analysis.

A total of 260 studies were screened, of which 8 studies met the inclusion criteria through direct screening, and an additional two studies were identified through systematic reviews, yielding a final total of ten studies included in this review. A PRISMA flow diagram detailing the study selection process is provided in Figure 1 [25].

### 3.1. Study Characteristics

Ten studies met inclusion criteria, spanning multiple surgical fields including aesthetic, orthopedic, otolaryngology, and burn surgery. Four were randomized controlled trials, three were prospective within-subject comparative studies, two were retrospective cohorts, and one was a prospective case series without a control group. Across all studies, TXA was applied topically or via subcutaneous infiltration in combination with epinephrine. Reported outcomes included intraoperative blood loss, blood loss per unit area, decantation ratio, ecchymosis, drain output, operative time, surgeon satisfaction scores, transfusion requirements, hemoglobin/hematocrit drop, Boezaart scores, and complications such as hematoma, thromboembolic events, and delayed wound healing. A summary of each study is provided in Table 1.

In a prospective observational study with a within-patient control design involving 38 patients undergoing burn wound excision and grafting, the control sites were treated with topical adrenaline solution alone (1:200,000), while the TXA-treated sites received a combination of adrenaline (1:200,000) and tranexamic acid (1000 mg in 200 mL normal saline), both applied using soaked sponges for 5–10 min to achieve hemostasis. The study found that TXA plus epinephrine significantly reduced mean blood loss per unit area (mL/cm^2^) across the upper limbs, lower limbs, and trunk, without compromising graft take or increasing complications [26].

In a retrospective cohort study of 76 patients undergoing rhytidectomy (32 control, 44 TXA), the intervention group received TXA at a final concentration of 9.1 mg/mL, added to both local and tumescent anesthetic mixtures containing varying concentrations of epinephrine (1:100,000 to 1:600,000). These solutions were injected along incision lines, anticipated dissection boundaries, and into the sub–superficial muscular aponeurotic system (SMAS) plane. The TXA group had significantly lower postoperative drain output on POD1 (14.8 cc vs. 50.4 cc; *p* < 0.001), with more frequent same day drain removal. Additionally, 75% of TXA patients experienced intraoperative blood loss < 50 cc compared to 25% in the control group (*p* < 0.001). One patient in the TXA group developed a postoperative pulmonary embolism, but no other significant differences in complications were observed [27]. In a retrospective case series of 23 patients undergoing rhytidectomy, TXA (1.5 mL of 100 mg/mL solution) was diluted into 150 mL of 0.5% lidocaine with 1:200,000 epinephrine for a final concentration of 1 mg/mL. A total of 60 mL of this solution was injected subcutaneously into the face and neck prior to incision. The authors reported a subjectively drier operative field compared to their prior experience using epinephrine alone and noted a marked reduction in hemostasis time, averaging approximately 6–7 min per side. No postoperative complications were observed [28].

In aesthetic surgery, a randomized, prospective within-subject study of 33 patients undergoing bilateral flank liposuction compared bruising in areas treated with TXA-containing tumescent solution versus control. Each flank received 500 mL of tumescent solution containing lidocaine, bupivacaine, and epinephrine; TXA (0.1%, 500 mg) was added to the solution on one side. The TXA-treated flanks showed significantly smaller bruising areas on postoperative days 1 and 7 compared to the control side [29]. In a randomized, double-blind trial of 36 patients undergoing bilateral breast liposuction, each breast was infiltrated with either a TXA-containing or control tumescent solution. All patients also received a 0.5 g intravenous bolus of tranexamic acid (TXA) at induction. The TXA tumescent solution contained 0.5 g TXA (0.5 g/5 mL) with epinephrine 1:100,000 per liter of saline, while the control solution contained epinephrine alone. The TXA group showed a 38% reduction in the ratio of decanted blood to total lipoaspirate (*p* = 0.0002), and dermal bleeding appeared improved on blinded video analysis. However, blinded photo assessment at 24 h postoperatively revealed increased ecchymosis in the TXA-treated breasts [30].

In Septoplasty, a double-blinded randomized trial (*n* = 60) compared local infiltration of lidocaine with epinephrine 1:100,000 versus the same solution combined with 100 mg of TXA. Both solutions were injected directly into the surgical field by the operating surgeon. The TXA group had significantly lower intraoperative blood loss (mean ~187 mL vs. ~341 mL), improved Boezaart surgical field scores (1.8 vs. 2.5), fewer additional hemostatic injections, and shorter operative times (44 vs. 56 min) compared to the epinephrine-only group [31].

In a randomized controlled trial of 90 patients undergoing bilateral total knee arthroplasty, a multi-step topical TXA protocol—including soft tissue infiltration, intra-articular injection, and bone sealing with TXA and epinephrine—was compared to epinephrine alone. The TXA group had significantly lower calculated total blood loss (693 mL vs. 953 mL; 27% reduction, *p* < 0.01) and a markedly lower transfusion rate (14% vs. 70%; RR 14.54, 95% CI: 5.01–42.18). Other outcomes, such as intraoperative visible blood loss, postoperative drain volume, and perioperative changes in hemoglobin and hematocrit, also favored the TXA group. There were no statistically significant differences in postoperative complications or knee function scores between groups [32].

A retrospective cohort study of 388 hips undergoing primary total hip arthroplasty compared outcomes between patients who received a TXA–epinephrine cocktail and those who received epinephrine alone. The TXA cocktail, which also contained local anesthetic, antibiotics, and morphine, was administered intraoperatively via a combination of soft tissue injection and intra-articular infiltration following fascial closure. Of the total cohort, 154 hips were treated with TXA, while 234 served as controls. The TXA group demonstrated significantly lower hemoglobin drop (mean 2.3 g/dL vs. 3.2 g/dL), estimated blood loss (695 mL vs. 819 mL), and transfusion rate (17% vs. 35%; *p* < 0.01). No significant differences were observed between groups in baseline characteristics, surgical parameters, or hospital stay [33].

A randomized, within-subject split-body trial of 40 patients undergoing functional endoscopic sinus surgery compared the effects of topical TXA (10 mg/mL), epinephrine (1:10,000), and their combination on intraoperative bleeding and surgical field quality. In Group I, TXA + epinephrine significantly reduced blood loss (35.4 vs. 53.5 mL) and improved field visibility compared to epinephrine alone. In Group II, epinephrine alone outperformed TXA alone in both field quality and operative time [34].

Lastly, a double-blind randomized controlled trial of 30 patients undergoing external dacryocystorhinostomy (DCR) compared intraoperative bleeding between gauze soaked with TXA (100 mg/mL) plus epinephrine (1:200,000) and gauze soaked with epinephrine alone. Fifteen patients were randomized to each group. The TXA + epinephrine group had significantly lower blood loss (29.4 ± 17.1 mL vs. 49.1 ± 18.1 mL; *p* = 0.005), fewer gauzes used (2.4 vs. 4.2; *p* = 0.008), and shorter operative time (36.0 vs. 46.1 min; *p* = 0.01). Surgeon satisfaction scores were higher in the TXA group, though not statistically significant [35].

### 3.2. Risk of Bias Across Studies

Among the ten included studies, four randomized trials were rated as having low overall risk of bias, with minor concerns primarily related to allocation concealment or potential unreported confounders [30,31,32,35]. Two additional randomized studies were assessed as having moderate risk due to variability in patient characteristics and possible systemic absorption of TXA [27,28,29]. Of the four non-randomized studies, one retrospective cohort [33] and one prospective observational study with a within-patient control [26] were assessed as having moderate overall risk of bias, largely due to limited control for confounding and incomplete reporting of patient variables. A second retrospective cohort was assessed as having serious risk of bias due to absence of blinding, unclear allocation criteria, and incomplete reporting of confounding variables [27]. One retrospective case series lacked a control group, objective outcome measurement, and consistent follow-up, and was rated at critical risk of bias [28].

Overall, the included studies varied substantially in study design and quality. Common limitations included lack of blinding, inconsistent reporting of baseline characteristics, absence of prespecified outcome definitions, and varied follow-up durations. Table 2 provides detailed risk of bias assessments for each included study.

## 4. Discussion

In this systematic review, we evaluated the addition of topically or locally applied tranexamic acid (TXA) to epinephrine across diverse surgical procedures, including burn wound excision, rhytidectomy, liposuction, septoplasty, total joint arthroplasty, facelift surgery, functional endoscopic sinus surgery, and external dacryocystorhinostomy [26,27,28,29,30,31,32,33,34,35]. Our findings highlight several key themes regarding the use of TXA combined with epinephrine in surgery. Across these settings, the combination was consistently associated with improved bleeding control, demonstrated by reductions in intraoperative blood loss, transfusion requirements, hemoglobin drop, gauze use, and early postoperative drain output, as well as better surgical field visibility and overall hemostasis. Several studies also suggested additional benefits, including shorter operative times and higher surgeon satisfaction, highlighting the broader clinical value of TXA used alongside epinephrine. Outcomes related to postoperative ecchymosis and bruising were less consistent, with some studies showing reductions and others reporting no difference or even greater bruising in the TXA group [29,30]. This variability may reflect differences in study design, anatomical site of surgery, outcome assessment methods, or timing of evaluation.

Concerns about thromboembolic side effects have historically limited use of TXA, yet the growing body of evidence continues to show its safety. Large trials and meta-analyses in non-cardiac and orthopedic surgery have consistently demonstrated reductions in blood loss and transfusion requirements without increased thromboembolic risk [36,37,38,39]. Our review supports these findings, as no major adverse events were directly attributable to TXA, aside from a single pulmonary embolism reported in one study, which was not considered causally related [27]. Synthesizing these results, TXA plus epinephrine emerges as an effective adjunct for perioperative bleeding control across surgical specialties, with a safety profile consistent with the broader TXA literature.

A major strength of this systematic review is its focus on a widely available, inexpensive, and long-standing antifibrinolytic agent—tranexamic acid (TXA)—whose use in surgery has not yet been optimized. Although TXA has been used for decades, its adoption remains inconsistent due to lingering concerns about thromboembolic complications, despite strong evidence demonstrating that such events are rare and that TXA is both safe and cost-effective [36,37,38]. Highlighting topical administration further strengthens this review, as it offers localized hemostatic benefit with minimal systemic exposure, maximizing safety while maintaining efficacy. Another strength is the focus on co-administration with epinephrine, which is already a standard part of perioperative practice in most surgical specialties. Evaluating TXA in this commonly used context underscores the practicality and direct clinical relevance of the findings. This review also benefits from its broad scope. By imposing no restrictions on surgical type, it allowed assessment of TXA across multiple specialties and organ systems, supporting the potential for wide extrapolation of results. Furthermore, the inclusion of diverse study designs, ranging from randomized controlled trials to prospective and retrospective cohort studies, as well as within-subject comparisons, provided a more comprehensive synthesis and improved generalizability. To our knowledge, this is among the first systematic reviews to specifically evaluate the combination of TXA and epinephrine, rather than TXA alone, thereby addressing a clinically important but previously underexplored question.

Despite these strengths, several important limitations must be acknowledged. While including multiple surgical specialties increases the breadth and relevance of our findings, it also introduces substantial heterogeneity. The procedures varied widely in design, dosing regimens, delivery methods, and outcome measures, which prevented us from combining results cumulatively or performing a more robust pooled analysis. Similarly, our inclusive approach to study design captured a wide body of evidence, but many of the retrospective or uncontrolled studies were judged to have a high risk of bias in our quality assessment. This inconsistency in study quality makes it more difficult to draw firm conclusions. A major limitation is the lack of adequately powered randomized controlled trials directly assessing topical or locally applied TXA in combination with epinephrine. Much of the existing evidence is drawn from small sample sizes, retrospective cohorts, or within-subject comparisons, which can provide useful preliminary insights but do not carry the same weight as well-designed clinical trials. Moreover, long-term safety outcomes were rarely reported, and adverse events were inconsistently defined across studies. Evidence for topical TXA in burn surgery remains particularly sparse, as most prior research in that field has focused on intravenous administration [40,41,42,43]. Taken together, these limitations underscore the need for more rigorous and standardized trials before widespread adoption of this approach can be confidently recommended.

Future research should prioritize adequately powered randomized controlled trials evaluating topical and locally applied TXA in combination with epinephrine across surgical specialties. Standardized protocols for dosing, delivery methods, and outcome measures are needed to enable meaningful comparison between studies. Trials that directly compare topical versus intravenous administration would also be valuable to clarify relative efficacy and safety. In addition, studies should incorporate long-term safety data, consistent adverse event reporting, and patient-reported outcomes to better capture the full clinical impact of TXA use.

## 5. Conclusions

This systematic review demonstrates that the addition of topically or locally applied tranexamic acid to epinephrine appears to reduce perioperative blood loss across a variety of surgical procedures, including aesthetic, orthopedic, otolaryngology, and burn surgery. Most studies reported improvements in bleeding-related outcomes—such as blood loss, drain output, operative field quality, and hemostasis time—without an associated increase in adverse events. Despite heterogeneity in study design, TXA dosing, and delivery techniques, the combined use of TXA and epinephrine consistently showed clinical benefit with a favorable safety profile. Based on current evidence, this combination appears to be a practical and potentially effective strategy for perioperative bleeding control, though further high-quality trials are needed before firm recommendations can be made.

## Figures and Tables

**Table 1:** Summary of included studies evaluating the use of topical or locally infiltrated tranexamic acid (TXA) in combination with epinephrine (epi) across various surgical procedures. Key study characteristics include procedure type, study design, sample size, TXA dosing and delivery method, comparator group (if applicable), reported outcomes, and complications. References for all studies listed are cited in the manuscript text. Abbreviations: TXA = tranexamic acid; NS = normal saline; EBL = estimated blood loss; Hb = hemoglobin; Hct = hematocrit; OR = operating room; CTBL = calculated total blood loss; epi = epinephrine; POD = postoperative day; PE = pulmonary embolism; DVT = deep vein thrombosis, DCR = dacryocystorhinostomy.

Study	Procedure Type	Study Design	Sample Size	TXA Dose and Route	Comparator	Outcomes Reported	Key Findings	Complications
Mohan et al., 2021 [26]	Burn wound excision	Prospective observational with within-patient control	38	1000 mg TXA in 200 mL NS (0.5%) with 1:200,000 epinephrine; applied topically via soaked sponge after excision	Epinephrine alone (1:200,000)	Blood loss per unit area, total blood loss, graft take, complications	TXA + epinephrine reduced blood loss by 36% compared to epinephrine alone; no impact on graft take	None reported
Schroeder et al., 2020 [27]	Rhytidectomy	Retrospective cohort	76 (44 TXA, 32 control)	TXA 9.1 mg/mL added to local anesthetic and tumescent; injected subcutaneously and into the sub-SMAS plane	Same anesthetic solutions with epinephrine, without TXA	POD1 drain output, days to drain removal, intraoperative EBL, hematoma, thromboembolic events	TXA reduced POD1 drain output (14.8 vs. 50.4 cc), earlier drain removal, and lower EBL; no difference in hematoma rate	1 PE in TXA group, no significant difference overall
Couto et al., 2020 [28]	Facelift (extended SMAS and SMAS plication)	Retrospective case series	27	1.5 mL TXA (100 mg/mL) in 150 mL lidocaine + epinephrine; subcutaneous injection	None	Time to hemostasis, estimated surgical time saved, subjective field dryness	TXA reduced time to hemostasis; improved field dryness; estimated 25–60 min surgical time saved	Minor skin healing delay (7.4%), 1 temporary neuropraxia (3.7%)
Fayman et al., 2021 [29]	Liposuction	Blinded prospective randomized case–control (within-patient)	33	500 mL tumescent solution with 0.1% TXA + epinephrine; injected into one flank	TXA-free tumescent solution (same patient)	Bruise area (day 1 and day 7)	TXA significantly reduced bruise area on days 1 and 7	None reported
Abboud et al., 2021 [30]	Liposuction	Randomized double-blind within-patient controlled trial	36	5 mL IV TXA at induction + 5 mL TXA in 1L NS with epinephrine (370 mL avg infiltrated per breast)	Epinephrine alone (same-patient control)	Decantation ratio, dermal bleeding, postoperative ecchymosis	TXA reduced decanted blood volume by 38% and intraoperative bleeding, but associated with increased ecchymosis	None reported
Hazrati et al., 2021 [31]	Septoplasty	Randomized double-blind controlled trial	60 (30 per group)	100 mg TXA in lidocaine + epinephrine; locally injected at surgical site	Lidocaine + epinephrine only	Blood loss, Boezaart score, satisfaction, operative time, hemodynamics	TXA group had significantly less blood loss, better scores, higher satisfaction, shorter OR time	None reported
Zhaohui et al., 2014 [32]	Bilateral total knee arthroplasty	Randomized controlled trial	90 (43 TXA, 47 control)	Multi-step: TXA + epinephrine soft tissue infiltration, intra-articular injection, bone sealing, drain clamping	Epinephrine-only infiltration; no TXA, no bone sealing; drain clamping	Blood loss, drainage, Hb/Hct drop, transfusions, swelling, function, adverse events	TXA group had 27% less blood loss, fewer transfusions, improved Hb/Hct, no significant diff in complications	TXA: 2 DVTs, 2 blisters, 3 bruises; Control: 3 DVTs, 3 hematomas, 2 blisters, 4 bruises
Chang et al., 2014 [33]	Total hip arthroplasty	Retrospective cohort	388 hips (154 TXA, 234 control)	10 mL of 5% TXA in local cocktail with epinephrine, anesthetics, antibiotics; injected intraarticularly	Cocktail without TXA	Hb drop, blood loss, transfusion rate, transfusion volume, hospital stay	TXA group had reduced Hb drop, EBL (695 vs. 819 mL), and transfusion rate (17% vs. 35%)	1 PE in control group
Aziz et al., 2024 [34]	Functional Endoscopic Sinus Surgery (FESS)	Randomized within-subject split-body trial	40	10 mg/mL TXA + 1:10,000 epinephrine; pledgets applied intranasally for 10 min	Epinephrine-only pledgets and TXA-only pledgets (same patient, opposite side)	Intraoperative blood loss, Boezaart score, surgery duration	TXA improved surgical field score, reduced blood loss, with shorter surgery duration	None reported
Salamah et al., 2023 [35]	External DCR	Double-blind randomized controlled trial	30	100 mg/mL TXA + epinephrine; gauze soaked and applied topically for 2 min	Epinephrine only	Intraoperative blood loss, surgery duration, gauze use, satisfaction	TXA significantly reduced blood loss, gauze use, surgical time	None reported

**Table 2:** Risk of bias assessment across included studies using the Cochrane RoB-2 and ROBINS-I tools, adapted for unified domain evaluation. Domains assessed included confounding, participant selection, intervention classification and blinding, deviations from intended interventions, missing data, outcome measurement, selective reporting, and overall risk of bias. Risk levels were categorized as Low, Moderate, Serious, or Critical. Domain-specific justifications are provided in the “Notes” row. References for all included studies are cited in the manuscript.

Risk of Bias Domain	Description	Mohan et al., 2021 [26]	Schroeder et al., 2020 [27]	Couto et al., 2020 [28]	Fayman et al., 2021 [29]	Abboud et al., 2021 [30]	Hazrati et al., 2021 [31]	Zhaohui et al., 2014 [32]	Chang et al., 2014 [33]	Aziz et al., 2024 [34]	Salamah et al., 2023 [35]
Confounding/Randomization	Were confounders adjusted or were groups randomized and balanced at baseline?	Moderate	Serious	Critical	Serious	Low	Low	Moderate	Low to Moderate	Low	Low
Participant Selection/Allocation Concealment	Were participants selected appropriately and/or was allocation concealed?	Moderate	Moderate to Serious	Moderate to Critical	Critical	Low	Moderate	Moderate	Moderate	Low	Low
Intervention Classification/Blinding	Was intervention clearly defined and were participants and researchers blinded?	Low to Moderate	Low to Moderate	Low	Moderate	Low	Low	Moderate	Low	Moderate	Low
Deviations from Intended Interventions	Were interventions implemented as planned (including intention-to-treat)?	Moderate	Moderate	Critical	Serious	Low	Low	Moderate	Low	Low	Low
Missing Data	Was follow-up complete or were missing data appropriately handled?	Low	Low	Serious	Critical	Low	Low	Low	Low	Low	Low
Measurement of Outcomes	Were outcomes measured objectively and were assessors blinded?	Moderate to Serious	Serious	Critical	Low to Moderate	Moderate	Low	Low	Low to Moderate	Moderate	Moderate
Selective Reporting	Were all pre-specified outcomes reported?	Moderate	Low to Moderate	Low	Moderate	Moderate	Low	Low	Low to Moderate	Low	Low
Overall Risk of Bias	Final judgment based on the above domains	Moderate	Serious	Critical	Serious	Low	Low	Low to Moderate	Moderate	Moderate	Low
Notes	Justifications or key limitations	Within-patient design minimize confounding. Definitions of outcomes were vague, and measurement methods lacked full objectivity.	Historical control design. All female participants. Most outcomes measured objectively but thromboembolic surveillance lacked detail.	No control group or objective measurements of outcomes. Bleeding was assessed subjectively by unblinded staff.	No information on initial sample size or loss to follow-up; systemic TXA absorption may have blurred control vs. experimental effects.	Strong RCT design with within-patient controls.	Well-executed RCT. Minor concerns remain regarding allocation concealment and unreported confounders.	Randomization method and allocation concealment were not described, raising risk of selection bias. concerns persist due to lack of blinding and unspecified exclusions.	Historical control design introduces time-based confounding risk. No blinding was mentioned, and VTE was not systematically screened.	Split-body randomized design minimized confounding, and interventions were clearly defined and applied consistently. However, lack of assessor blinding for subjective outcomes introduced moderate detection bias.	Well-conducted double-blind RCT with clearly defined interventions and complete follow-up.

## Appendix A. Search Strategies

**Table:**

Database	Search Strategy
MEDLINE Ovid MEDLINE(R) ALL 25 June 1946 to 2025	(tranex?mic or TXA or cyklokapron or cyklo-F).mp. (epinephrine or adrenalin*).mp. (topical or soak* or gauze*).mp. 1 and 2 and 3
Embase Embase 25 June 1974 to 2025	(tranex?mic or TXA or cyklokapron or cyklo-F).mp. (epinephrine or adrenalin*).mp. (topical or soak* or gauze*).mp. 1 and 2 and 3
CINAHL	(tranexamic or tranexemic or TXA or cyklokapron or cyklo-F) AND (epinephrine or adrenalin*) AND (topical or soak* or gauze*)
Cochrane Library	(tranexamic or tranexemic or TXA or cyklokapron or cyklo-F) AND (epinephrine or adrenalin*) AND (topical or soak* or gauze*)
Scopus	(tranexamic or tranexemic or TXA or cyklokapron or cyklo-F) AND (epinephrine or adrenalin*) AND (topical or soak* or gauze*)
Web of Science Core Collection	(tranexamic or tranexemic or TXA or cyklokapron or cyklo-F) AND (epinephrine or adrenalin*) AND (topical or soak * or gauze*)
ProQuest Dissertations & Theses Citation Index	(tranexamic or tranexemic or TXA or cyklokapron or cyklo-F) AND (epinephrine or adrenalin*) AND (topical or soak* or gauze*)
Google Scholar	(tranexamic OR tranexemic OR TXA OR cyklokapron OR cyklo-F) AND (epinephrine OR adrenalin*) AND (topical OR soak* OR gauze*)
